# The two decades brainclinics research archive for insights in neurophysiology (TDBRAIN) database

**DOI:** 10.1038/s41597-022-01409-z

**Published:** 2022-06-14

**Authors:** Hanneke van Dijk, Guido van Wingen, Damiaan Denys, Sebastian Olbrich, Rosalinde van Ruth, Martijn Arns

**Affiliations:** 1 Research Institute Brainclinics, Brainclinics Foundation, Nijmegen, the Netherlands; 2 Faculty of Psychology & Neuroscience, Department of Cognitive Neuroscience, Maastricht University, Maastricht, the Netherlands; 3 Amsterdam UMC, Department of psychiatry, University of Amsterdam, location AMC, Amsterdam, the Netherlands; 4 Department for Psychiatry, Psychotherapy and Psychosomatics, University Hospital of Psychiatry Zurich, Zurich, Switzerland; 5 neuroCare Group, Nijmegen, the Netherlands

**Keywords:** Neurophysiology, Psychiatric disorders, Biomarkers, Translational research, Prognosis

## Abstract

In neuroscience, electroencephalography (EEG) data is often used to extract features (biomarkers) to identify neurological or psychiatric dysfunction or to predict treatment response. At the same time neuroscience is becoming more data-driven, made possible by computational advances. In support of biomarker development and methodologies such as training Artificial Intelligent (AI) networks we present the extensive Two Decades-Brainclinics Research Archive for Insights in Neurophysiology (TDBRAIN) EEG database. This clinical lifespan database (5–89 years) contains resting-state, raw EEG-data complemented with relevant clinical and demographic data of a heterogenous collection of 1274 psychiatric patients collected between 2001 to 2021. Main indications included are Major Depressive Disorder (MDD; N = 426), attention deficit hyperactivity disorder (ADHD; N = 271), Subjective Memory Complaints (SMC: N = 119) and obsessive-compulsive disorder (OCD; N = 75). Demographic-, personality- and day of measurement data are included in the database. Thirty percent of clinical and treatment outcome data will remain blinded for prospective validation and replication purposes. The TDBRAIN database and code are available on the Brainclinics Foundation website at www.brainclinics.com/resources and on Synapse at www.synapse.org/TDBRAIN.

## Abstract (summary)

**Table:** 

Measurement(s)	psychiatric and healthy resting state EEG
Technology Type(s)	electro-encephalography (EEG)
Sample Characteristic - Organism	human
Sample Characteristic - Environment	laboratory environment
Sample Characteristic - Location	Province (The Netherlands)

## Background & Summary

The human electroencephalogram (EEG) was first described almost a 100 years ago by Hans Berger^1^. EEG activity arises from the summation of electrical potentials of thousands of synchronously active post-synaptic (inhibitory as well as excitatory) currents of aligned pyramidal cells and has a temporal resolution of milliseconds. Since its discovery, many studies have used EEG to investigate the neurophysiological underpinnings of various kinds of human capacities in research laboratories, and dysfunctions in clinical settings aiming to improve mental health treatments.

In applied neuroscience research, EEG data are often used to extract features, also called biomarkers, that can identify a certain neurological or psychiatric diagnosis or predict response to a specific treatment to improve treatment decisions. Many biomarker-studies often employ statistically underpowered sample sizes, and lack validation or replication^2,3^. As a result, meta-analyses have failed to confirm some of the most well-known biomarker findings such as frontal alpha asymmetry (FAA) in MDD^4^ or theta-beta ratio (TBR) in ADHD^5^. Furthermore, a recent meta-analysis on EEG-biomarkers predicting MDD treatment response, concluded that those investigated were generally not reliable due to a strong publication bias and a lack of out-of-sample validation and replication studies^4^. These conclusions have been followed-up by initiatives, such as the ICON-DB consortium that aims to make EEG data from repetitive Transcranial Magnetic Stimulation (rTMS) studies available for direct replication. The ICON-DB consortium initiative already resulted in a published non-replication^6^ and a successful replication^7^.

A promising development in EEG research is the use of artificial intelligence (AI) as an advanced signal processing tool, for example to define EEG characteristics that could identify sex^8^, neurological EEG pathology^9,10^, or response to different types of therapy^11^. To successfully employ AI techniques (e.g., machine-learning or deep-learning) one should prevent overfitting since this commonly leads to a lack of generalization and therefore negates the applicability of the specific AI model. To do this, the total dataset should be sub-divided into training-, validation- (together used to develop a model) and an independent and separately held test-sets (to test the generalizability). Therefore, it is well known that a large amount of data is imperative. Unfortunately, the literature is scant of EEG-AI studies where no test-sets are used and/or small samples of N < 50 (or not reported) without cross-validation, where accuracies of >90% are claimed (for reviews see^12,13^). In support of the development of robust biomarkers, as well as new methodologies in applied neuroscience, we here present a large single-site, standardized raw EEG lifespan database (N = 1274, 620 female, age 38.67 ± 19.21 (range 5–88) years, and a total of 1346 EEG sessions, including a replication sample) of a heterogeneous sample of healthy- as well as psychiatric participants with a variety of psychiatric patients. Major disorders of the database are MDD (N = 426), ADHD (n = 271), SMC (n = 119) and OCD (n = 75), for which well characterized treatment-outcome data have been published before. The database consists of both baseline and multiple session (full time-series, raw) EEG recordings collected over a period of two decades as part of routine clinical care and applied neuroscience projects (see Table 1 for studies published on this dataset) in a single EEG lab. It contains data to investigate or replicate both diagnostic (ADHD, MDD, OCD) as well as prognostic biomarkers (rTMS, neurofeedback). In addition to the raw EEG recordings, the TDBRAIN database also contains autonomic measures such as electro-cardiography (ECG, which is measured with the same device), and behavioral data from an auditory oddball task as well as a visual 1-back task. Moreover, demographic and clinical data, such as gender, age, height, weight, sleep, education, alcohol, drug use, and item level NEO-FFI (Big-five personality questionnaire) data are available in the database. For the published data the clinically relevant data such as primary outcome measures and details on the neuromodulation parameters are included. Neurophysiological quality of the data was validated based on two well-known phenomena; (1) alpha oscillatory power attenuates from closing to opening the eyes^1,14^ and (2) the maturational change in peak frequency of these alpha oscillations (iAPF) from childhood to adulthood^15–17^.

**Table 1:** Number of sessions per indication and formal diagnosis (Dx) * of 176 participants included in^23^, ** of which all included in^25^, *** of 16 participants included in^27^, **** Includes small samples of: Migraine, PDD NOS, Anxiety, Depersonalization, Conversion, ASD, Asperger, TBI, Bipolar disorder, Whiplash and Dyspraxia.

Indication	No. EEG Sessions	with Formal Dx
MDD	426	198*
ADHD	271	141**
SMC	119	
OCD	75	58***
Tinnitus	33	
Insomnia	32	32
Parkinson	27	17
Burnout	10	10
Dyslexia	26	20
Chronic Pain	14	14
Other****	80	
UNKNOWN	255	
Healthy	47	

Note, participants can have multiple indications.

## Methods

EEGs were recorded in accordance with the standardized methodology as developed by Brain Resource Ltd. (details of which can be found here^18^), of which reliability, validity, and across site-consistency has been published elsewhere^19–21^. The data of all participants included in the database was recorded as part of treatment-as-usual, and all participants provided informed consent stating *“…. I agree that scientists can have access to this data at any time in the future and that the data may be used for any scientific, clinical or commercial purpose. I also understand that any information that personally identifies me/ my son / my daughter, is NOT part of the database and is confidentially and separately stored from my/her/his brain data…”*, which was manually verified before including the participant in the currently presented TDBRAIN database. Participants were asked to wash their hair with shampoo without conditioner and not use hairstyling products like gels on the day of measurement. In addition, participants were asked to refrain from the use of alcohol for 6 hours before the EEG assessment, smoke as little as possible on the day of the assessment and not to smoke and drink beverages with caffeine for two hours before the assessment. Medication usage was allowed – but not systematically tracked - and patients on psychoactive medication with short halve-lives (e.g methylphenidate) were encouraged to skip the morning dosage before the EEG assessment.

**Fig. 1:**
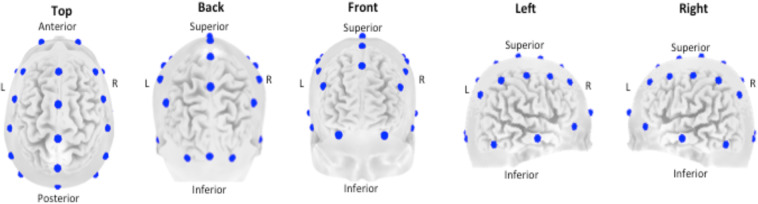
Electrode positions (blue dots) shown from different perspectives: Top, back, front, left and right views. For exact position coordinates (x,y,z) see Table 3.

During set-up for EEG recordings, participants answered questions on two questionnaires which pertain to their recent activities and the NEO-FFI which identifies scores on five distinct personality traits: Neuroticism, Extraversion, Openness, Agreeableness and Conscientiousness. Psychophysiological recordings include 26 channel EEG-recordings, based on the 10–10 electrode international system (see Fig. 1) using a Compumedics Quickcap or ANT-Neuro Waveguard Cap with sintered Ag/AgCl electrode, acquired at a sampling rate of 500 Hz (low-pass filtered at 100 Hz prior to digitization). The EEG was recorded with a virtual ground and offline referenced to averaged mastoids (A1 and A2) with a ground at AFz and skin resistance was kept below 10 kΩ using a conductive non-toxic aqueous gel (Quick-Gel, conductive gel, Compumedics NeuroMedical Supplies, USA or OneStep Cleargel). Vertical- and horizontal eye movements were recorded with electrodes placed 3 mm above the left eyebrows and 1,5 cm below the left bottom eye-lid, and 1.5 cm lateral to the outer canthus of each eye respectively. In addition, the ECG, measured at the clervical bone (Erbs) as well as the electromyogram (EMG, at the right masseter muscle) were recorded (see Table 3 for a complete overview). Data were assessed during resting state, consisting of: a 2-minute Eyes Open (EO) task, where the subject was asked to rest quietly, with eyes open and focus on the red dot at the center of the computer screen in front of them, and a 2-minute Eyes Closed (EC) task, where the subject was asked to close their eyes and retain the same position as before. Behavioral measures (reaction-times and responses) are included for an auditory oddball task and a visual 1-back memory task, that were performed after the resting state conditions. For the oddball task participants were presented with a series of low- (500 Hz) and high- (1000 Hz) pitched tones (50 ms, 75 dB) with an interstimulus interval (ISI) of 1 s. Participants were instructed to respond to the high pitched ‘target’ tone (60 targets out of 340 stimuli) with both index-fingers. In the visual 1-back task, letters (B, C, D and G) were presented at the center of the screen for 200 ms with an ISI of 2.5 s. Participants were instructed to respond with both index-fingers when a letter was the same as the previous letter (20 targets out of 125 stimuli).

## Data Records

**Fig. 2:**
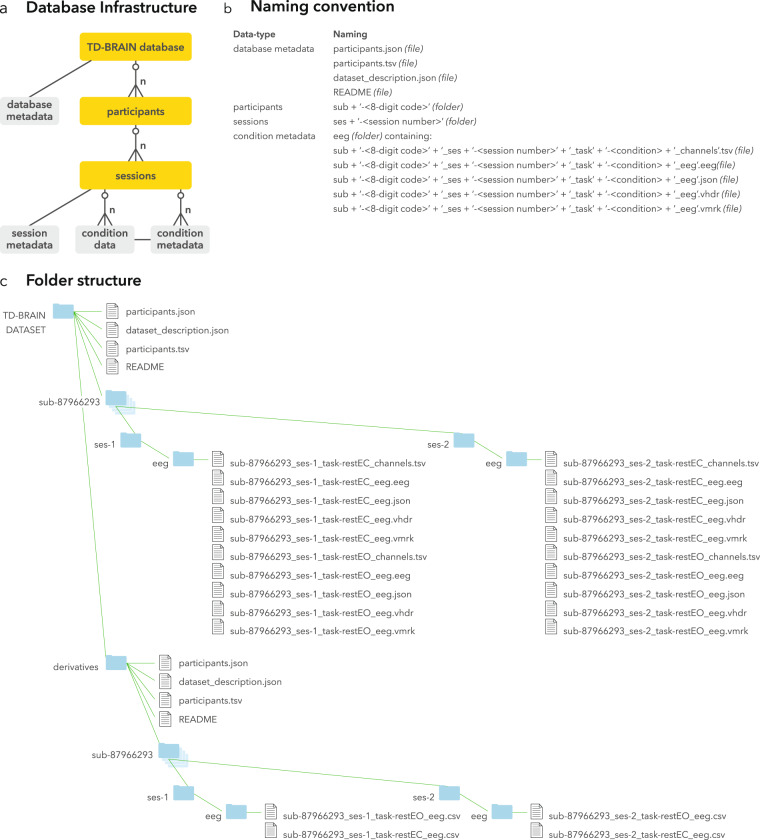
Database design and naming convention. (**a**) shows the infrastructure, the TDBRAIN consists of a file containing the participants metadata and multiple participants folders, these in turn may include multiple session folders. In the session folders, session specific information is stored in the session metadata, the condition files (EEG data) measured within this session are stored as.csv files and their specific information in condition metadata. (**b**) the naming convention: participants always have 8 digit IDcodes, sessions are described with the participants IDcode and then ‘-’ + <sessionnumber>. Each EEG measurement additionally acquires a condition, such as ‘.EO’ or ‘.EC’ in the current database. These measurements possibly will be complimented with several additional conditions, with condition having a maximum of 4 characters. (**c**) shows an example of one participants’ folder and file structure.

**Fig. 3:**
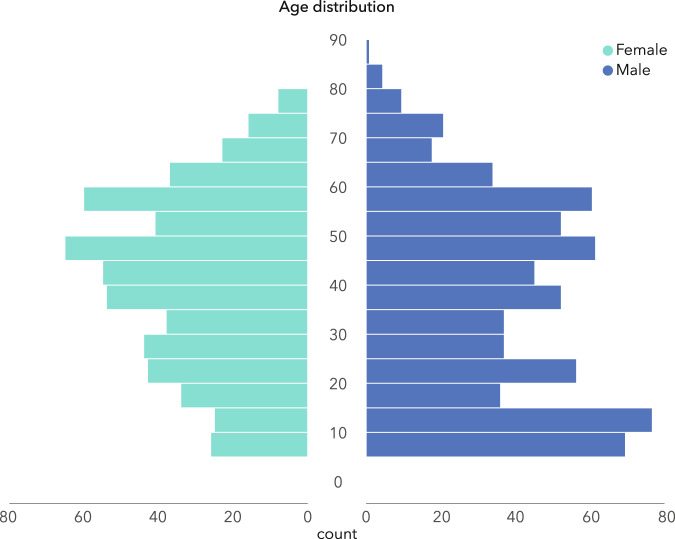
Age distribution for female (green) and male (blue) participants, for the whole heterogenous database.

The entire dataset (n = 1274; TD-BRAIN-DATASET) as well as a smaller trial-set (n = 20; TD-BRAIN-SAMPLE) and the complementary custom python code, can be found as split-zip files on the Brainclinics Foundation website at www.brainclinics.com/resources and in parallel on the data repository Synapse at www.synapse.org/TDBRAIN (10.70303/syn25671079)^22^. On www.brainclinics.com/resources it will be required to login through ORCID and sign a Data Use Agreement (see Supplement S1-DUA-BCResources.pdf) after which the dataset as well as a complementary custom python code (10.70303/syn25671079^22^) used for preprocessing (which was reviewed and beta-tested) can be downloaded. For downloading via www.synapse.org one must be a registered user and agree with the same terms (see Supplement: S2-DUA-Synapse.pdf) in accordance with the European privacy rules (GDPR). Both repositories contain a README file that describes how to download and unpack the data (see Fig. 2 for an overview of the database structure, as well as the naming convention). Table 2 provides an overview of the data(sets) included in the TDBRAIN database, and Fig. 3 depicts the age distribution of male- and female participants separately. The database contains participants with a 1) formal diagnosis (Dx; DSM-5) confirmed by a licensed clinician and/or by a structured clinical interview and requiring exceeding a clinical cut-off, or 2) participants with a referral-indication, meaning an unofficial diagnosis the client was referred with for the EEG-assessment by a general practitioner or psychologist/psychiatrist. The database also includes clients missing this information for which the indication and Formal-Dx are marked with UNKNOWN. Note that this does not mean these are healthy participants. Most patients within the MDD sample received treatment with Dorsolateral Prefrontal Cortex (DLPFC) rTMS (n = 176^23^) and patients were included in the study with 1) a primary diagnosis of non-psychotic MDD or dysthymia, 2) Beck Depression Inventory (BDI-II-NL)^24^ >14 at baseline, 3) treatment with at least 10 sessions of rTMS over the DLPFC or response within these 10 sessions. Exclusion criteria for the rTMS sample were: prior ECT treatment, epilepsy, traumatic brain injury, a current psychotic disorder, wearing a cardiac pacemaker, metal parts in the head, or pregnancy. The QEEG-*informed* Neurofeedback ADHD (n = 102^25^) sample consists of patients that were 1) diagnosed with ADHD confirmed by the MINI Diagnostic Interview or by a qualified clinician 2) ADHD-RS^26^ scores on either scale (ATT or HI) were equal to or higher than 6 (for adults a cut-off of 5 or higher was used, in line with current DSM-5 diagnostic requirements. The Supplementary Motor Area (SMA) rTMS in OCD (n = 17^27^) sample includes patients that had a primary DSM-IV diagnosis of OCD based on the MINI International Neuropsychiatric Interview (MINI^28^) 2) had failed at least two previous treatments, 3) and completed at least 10 sessions of rTMS. Exclusion criteria were the same as above for the DLPFC-rTMS sample. An overview containing all participant-information and measurement-sessions are presented in Tables 1 and 2 respectively.

**Table 2:** Availability of demographic-, personality-, clinical- and measurement-day data.

		Nr. participants	Nr. EEG sessions
	total	1274	1346
Number of sessions	1	1205	
	2	65	
	3	4	
MDD	BDI (pre&post)	176	198
	rTMS protocol 1	65	76
	rTMS protocol 2	105	114
	rTMS protocol 3	7	8
ADHD	ADHDRS (pre&post)	102	102
OCD	YBOCS (pre&post)	48	48
Demographics per session	Age		1323
	Gender		1345
	Weight		593
	Height		593
	Education		1337
neoFFI	60 items		1018
Neuropsych. measurements	Oddball- and 1-back memory task measurements (Correct Positives, False Positives, Correct Negatives, False Negatives and reaction-times)		1297
Day of measurement	Day of measurement data (per session)		1345
	reported to have recently smoked		303
	reported recent alcohol consumption		406
	reported recent drug consumption		52
	Time of day (morning)		147 (48 AM/ 99 PM)
	Season		1320 (360 Winter, 338 Spring, 283 Summer, 339 Fall)

**Table 3:** Complete overview of all EEG electrodes and their positions, as well as the additional electrodes.

Electrodes	Full name/description	EEG Coordinates		
		X	Y	Z
Fp1	Frontopolar 1	−26.81	84.06	−10.56
Fp2	Frontopolar 2	29.41	83.74	−10.04
F7	Frontal 7	−66.99	41.69	−15.96
F3	Frontal 3	−48.05	51.87	39.87
Fz	Frontal zero	0.90	57.01	66.36
F4	Frontal 4	50.38	51.84	41.33
F8	Frontal 8	68.71	41.16	−15.31
FC3	Frontocentral 3	−58.83	21.02	54.82
FCz	Frontocentral zero	0.57	24.63	87.63
FC4	Frontocentral 4	60.29	21.16	55.58
T7	Temporal 7	−83.36	−16.52	−12.65
C3	Central 3	−65.57	−13.25	64.98
Cz	Central zero	0.23	−11.28	99.81
C4	Central 4	66.50	−12.80	65.11
T8	Temporal 8	84.44	−16.65	−11.79
CP3	Centroparietal 3	−65.51	−48.48	68.57
CPz	Centroparietal zero	−0.42	−48.77	98.37
CP4	Centroparietal 4	65.03	−48.35	68.57
P7/T5	Temporal 5	−71.46	−75.17	−3.70
P3	Parietal 3	−55.07	−80.11	59.44
Pz	Parietal zero	−0.87	−82.23	82.43
P4	Parietal 4	53.51	−80.13	59.40
P8/T6	Temporal 6	71.10	−75.17	−3.69
O1	Occipital 1	−28.98	−114.52	9.67
Oz	Occipital zero	−1.41	−117.79	15.84
O2	Occipital 2	26.89	−114.68	9.45
VPVA	Vertical positive vertical above			
VNVB	Vertical negative vertical below			
HOHL	Horizontal left			
HNHR	Horizontal right			
Erbs	ECG measured at the Clavicle bone			
OrbOcc	Orbicularis Oculi (between VNVB and HOHL)			
MASS	Masseter

All data (combined raw EEG recordings, demographic, clinical and behavioral) are organized in Brain Imaging Database Structure (BIDS)^29^ format and presented in BrainVision Analyzer (BVA) readable format as well as.csv format (in the derivatives folder). Each participants session-data contains two EEG files (EO and EC) in.eeg and.vhdr format, a.json file with recording information for each session and each condition, as well as the channel information in a.tsv file for each condition. Both the BIDS and derivatives folder contain participants.tsv and participants.json files containing the available information for all participants in one overview. The TD_BRAIN_code folder contains the python package used to analyze the.csv data (taken from the derivatives folder) and create the output described in this manuscript.

### Replication/validation repository

As previously described, in response to the replication crisis^3^, thirty percent of the known diagnostic and prognostic categories of the MDD, ADHD and OCD datasets will remain blinded confirming age, gender and response distributions are the same for the blinded data. Blinded data are characterized with a participant_id starting with ‘sub-19’ as well as with the REPLICATION in the participants.tsv file. Researchers are encouraged to share their predictions about diagnostic status or treatment response as well as their methods by submitting predicted group membership (diagnosis or responder/remitter) as well as methods to the Brainclinics Foundation (by an e-mail to the corresponding author using [TDBRAIN] in the subject), so accuracies can be established by independent verification against the diagnostic or prognostic data that is known to the corresponding author. Prediction accuracies will be disclosed to the researchers and be made available on the Brainclinics Replication/Validation repository (at www.brainclinics.com/resources), which will be available as source of verified independent replication that can be consulted by editors and peer-reviewers during the peer-review process, when the researchers have submitted their research and replication.

## Technical Validation

### Hardware

**Fig. 4:**
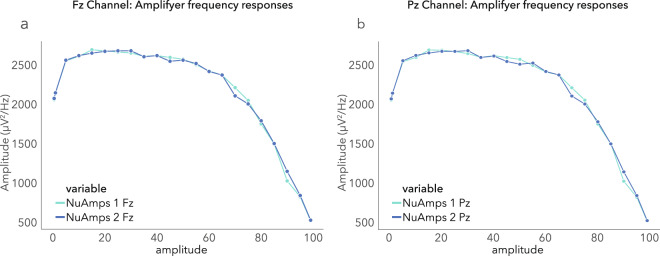
The frequency response of the two amplifiers used in this dataset for the two EEG channels, (**a**) Fz and (**b**) Pz.

The frequency response of two different Neuroscan NuAmps amplifiers used while recording the data were tested using a Neuroscan PocketTrace2 signal generator, and a sine wave with a 50 uV peak-to-peak amplitude was injected at 0.5, 1, 5, 10, 15, 20, 25, 30, 35, 40, 45, 50, 55, 60, 65, 70, 75, 80, 85, 90, 95 and 99 Hz at Fz and Pz referenced against A1. Twenty second segments at each frequency were extracted and the spectral peak determined in uV^2^/Hz. Figure 4 depicts these results and confirms the flat frequency response from 0–100 Hz as well as the similarity between channels (Fz and Pz) and the 2 different amplifiers. The tapering of around 80 Hz is the result of the low-pass filter used at 100 Hz.

### Neurophysiological validation

To guaranty the neurophysiological quality all data was manually checked. Moreover, to assess the usability for signal processing of the EEG measurements, two tests were performed, reflecting two well-known phenomena: (1) The power of alpha-band oscillations (7–13 Hz) should increase when participants have their eyes closed relative to when they have their eyes open^1,14^, (2) the frequency of alpha-band oscillations indicates EEG-maturation, increasing from 6–18 years old, then leveling out and decreasing at older ages^15–17^.

### Preprocessing

For the neurophysiological validation all data analysis was performed using relevant python modules, such as numpy^30^ and scipy^31^. To be able to pre-process and de-artifact large amounts of EEG datasets we adapted previously published automatic preprocessing routines to be compatible for use in python for subsequent digital signal-processing and artificial intelligence applications^18,32–34^ (see *code availability*). In short, the bipolar EOG was computed and removed from the EEG-signal using the method published by Gratton et. al.^32^. Data were demeaned and bandpass-filtered between 0.5 to 100 Hz and the notch-frequency of 50 Hz was removed. Following, various artifact signals were detected: (1) EMG, (2) sharp channel-jumps (up and down), (3) kurtosis, (4) extreme voltage swing, (5) residual eyeblinks, (6) electrode bridging^33^ and (7) extreme correlations. If a channels’ signal contained artifacts for more than 66% of the measurement it was repaired using a Euclidian distance weighted average of at least 3 neighboring channels. The resulting EEG data that was clean of artifacts was segmented into 5 second segments and used for subsequent analysis.

### Frequency analysis

#### Power difference between eyes open and eyes closed

**Fig. 5:**
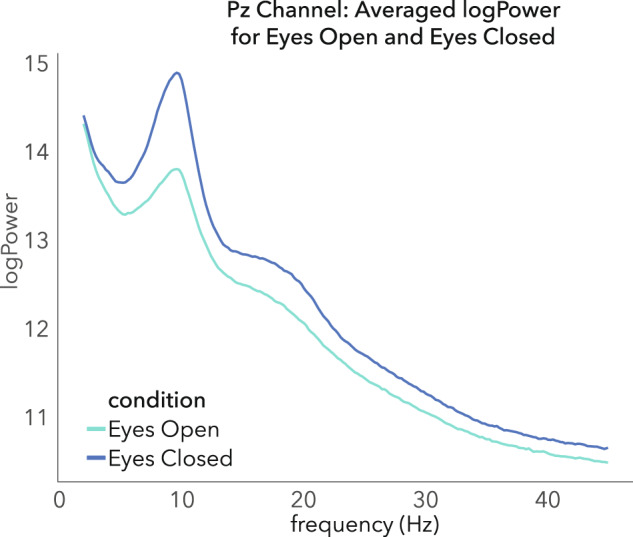
Averaged logPower measured at Pz, for Eyes Open (green) and Eyes closed (blue). The difference is significant between 7 and 13 Hz (p < 0.001, d’ = 0.9).

The power spectrum between 2 and 45 Hz was computed for the EEG electrode Pz in the EO and EC conditions separately, by using a Fast-Fourier Transform (FFT) on each 5 second segment convolved with a segment length Hann window and then normalized using a natural logarithm. The computed power-spectra for each segment were first averaged within participants, and then within conditions. All measurement sessions were included and a t-test for dependent samples was performed to compare EO and EC over the frequency range between 7 and 13 Hz. In line with the expectations the log-power of alpha oscillations (7––13 Hz) increased from Eyes Open (EO) to Eyes Closed (EC) with a large effect size (d’ = 0.89, p < 0.001). The results are depicted in Fig. 5 and show that the signal attenuates with opening the eyes.

#### Maturation of the iAPF

**Fig. 6:**
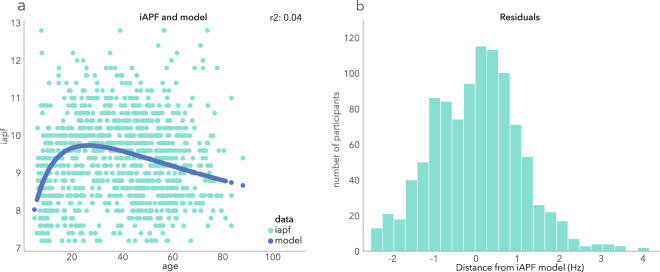
The iAPF (at Pz) related with age, and iAPF predicted from age. (**a**) the iAPF of all participants sorted by age (green) and the logGaussian function modeling the iAPF from age (blue). The model explains 4% of the variance and shows an initial steep increase of iAPF up till an age of approximately 18 years after which a slight decrease sets in. b) the distribution of the residuals that shows to be normal with a mean of 0.003 +/− 1.06 (Shapiro test for normality; stat = 0.99, p < 0.001).

Using the computed power spectra at electrode Pz we determined the individual alpha peak frequency (iAPF) between 7 and 13 Hz (using scipy.signal.find_peaks). For each subject, the peak with the maximum power and having a value of at least 40% (taking into account the 1/f signal) from the maximum power in the frequency range of interest and a difference of 0.05 uV^2^ with its neighboring frequency was defined as the iAPF. To assess the initial maturation related increase in iAPF up till approximately 18 years of age and following decrease in older ages, the iAPFs were sorted according to each subject’s age, and subsequently modeled using a logGaussian function which was optimized for the shape the data were hypothesized to show. As hypothesized the iAPFs show an initial steep increase up till approximately 18 years of age and subsequently show a slight decrease. The log-Gaussian model explained 4% of the variance (R^2^ = 0.04; Fig. 6a). And the resulting residuals were normally distributed with a mean of 0.003 +/− 1.06 (Shapiro test for normality; stat = 0.99, p < 0.001, Fig. 6b), indicating the model is a good fit.

## Usage Notes

These data can be instrumental in testing and validating diagnostic and prognostic psychiatric applications as well as to investigate lifespan patterns in EEG parameters and ANS phenomena such as heart rate, heart rate variability measures and eye blink rates, or the interrelation and interdependency between these domains (e.g., heart-beat evoked potentials). Furthermore, given the data are fully unprocessed data recorded between DC-100 Hz with full 24-bit resolution and availability of several artifact channels (EOG, EMG, ECG) these data can also be used to test, develop and validate new EEG pre-processing and de-artifacting routines.

## Supplementary information

DUA for www.brainclinics.com/resources

DUA for www.synapse.org/TDBRAIN

## Data Availability

The data presented in the database contains raw, full time-series EEG recordings and it is possible to analyse in any way. Nonetheless, for full transparency and replicability the complementary custom python code used for preprocessing (which was peer reviewed and beta-tested) as well as the code used for the neurophysiological validation is published together with the entire dataset on www.brainclinics.com/resources as well as on www.synapse.org (10.70303/syn25671079)^22^ in one package and available under the same conditions described above. In addition, we have also published the TD_BRAIN_code on github: https://github.com/BCD-gitprojects/TDBRAIN/.
