# Supplementary material for: The two decades brainclinics research archive for insights in neurophysiology (TDBRAIN) database
Source: Sci Data. 2022 Jun 14;9:333. doi: 10.1038/s41597-022-01409-z (PMC9198070; doi:10.1038/s41597-022-01409-z)
Supplement: Supplementary file 1 — DUA for www.brainclinics.com/resources [file 41597_2022_1409_MOESM1_ESM.pdf]

Data Use Agreement www.brainclinics.com/resources

Brainclinics Foundation

I request access to the TD-BRAIN dataset published in van Dijk et al. (*The Two Decades - Brainclinics Research Archive for Insights in Neurophysiology (TD-BRAIN) database*, Nature Scientific Data, 2021) via the website www.brainclinics.com/resources of the Brainclinics Foundation, Nijmegen, the Netherlands, and thereby agree to the following:

1) I will comply with all relevant rules and regulations imposed by my institution and my government. This possibly means my research needs to be approved or declared exempt by a committee that oversees research on human subjects, e.g. my Institutional Review Board or Ethics Committee.
2) I will not attempt to establish the identity of, neither attempt to contact any of the included human subjects nor link this data to any other database in a way that could result in identifiable information. I understand that under no circumstances will the key that would link these data to an individual's personal information be provided to me under these Data Use Terms.
3) I will not redistribute or share the data with others, including individuals in my research group, unless they have independently applied, approved this DUA and been granted access to this data via the www.brainclinics.com/resources.
4) I will not make changes to the raw data provided in the TD-BRAIN dataset.
5) I will give appropriate credit when using the data,
    a) by providing a link to www.brainclinics.com/resources and including a reference to the van Dijk et al. (2021) publication.
    b) Papers, book chapters, books, posters, oral presentations, and all other presentations of results derived from the data should acknowledge the origin of the data. Authors of publications or presentations using the data should cite relevant publications describing the methods developed and used by the Brainclinics Foundation to acquire and process the data. Neither the Brainclinics Foundation, nor the researchers that provide this data should be included as an author of publications or presentations if this authorship would be based solely on the use of this data.
6) The code is provided under the MIT open access license where you must give appropriate credit when using the code,
    a) by providing a link to www.brainclinics.com/resources and including a reference to the van Dijk et al. (2021) publication, indicating if changes were made when publicly presenting any results or algorithms that benefitted from their use.
    b) Papers, book chapters, books, posters, oral presentations, and all other presentations of results derived from the data should acknowledge the origin of the data. Authors of publications or presentations using the code should cite relevant publications describing the methods developed and used by the Brainclinics Foundation to process the data. Neither the Brainclinics Foundation, nor the researchers that provide this code should be included as an author of publications or presentations if this authorship would be based solely on the use of this code.
    c) If the material is remixed, transformed, or build upon, I will distribute my contributions under the same license as the original.

7) I do not have to comply with the license for elements of the material in the public domain or where my use is permitted by an applicable exception or limitation.
8) I understand that no warranties are given. The license may not give all of the permissions necessary for my intended use. For example, other rights such as publicity, privacy, or moral rights may limit how the material can be used.
9) Failure to abide by these guidelines will result in immediate termination of my privileges to access to these data.
